# Unveiling organohalide respiration potential in River Nile sediments via 16S rRNA gene amplicon sequencing of endogenous bacterial communities

**DOI:** 10.1186/s12866-025-03864-1

**Published:** 2025-03-31

**Authors:** Hwayda Soliman, Mohamed Ismaeil, Hoda Soussa, Wael S. El-Sayed

**Affiliations:** 1 Microbiology Department, Faculty of Science, Ain Shams University, Cairo, Egypt; 2 Irrigation and Hydraulics Department, Faculty of Engineering, Ain Shams University, Cairo, Egypt

**Keywords:** Biodegradation, River Nile sediment, Organohalide respiration, 16S rRNA, Functional biomarkers

## Abstract

**Background:**

Industrial waste, agricultural runoff and untreated sewage contaminate the Nile, leaving a toxic legacy in its sediments. Organohalides-polluted sediment in particular poses serious public health risks and detrimental effects on aquatic life. Sediment microbiomes may harbor bacterial strains that could be utilized in bioremediation of such toxic pollutants.

**Material and methods:**

Two microbiomes from polluted River Nile sediments were analyzed by using 16S rRNA gene amplicon sequencing. In addition, PICRUSt analysis based on 16S rRNA data was used to explore the organohalide respiring bacteria (OHRB) genera and their corresponding organohalide respiration (OHR) activity. Microcosm studies were performed to validate the potential for dechlorination activity of River Nile sediment. Dechlorination of the parent chloroethenes into daughter end product were detected by gas chromatography coupled with flame ionization detection analysis.

**Results:**

Analysis of 16S rRNA gene amplicon sequences using the EZ-biocloud server identified *Proteobacteria* as the dominant phylum in both microbiomes, with *Bacteroidetes* and *Chloroflexi* prevalent in RNS1 sediment and *Chlorobi* in RNS2 sediment. EZ-biocloud and PCR analyses detected several potential OHRB genera, including *Dehalococcoides*, *Dehalogenimonas*, *Desulfomonile*, *Desulfovibrio*, and *Geobacter*, suggesting potential OHR activity. Further evidence for potential OHR activity was provided by PICRUSt functional prediction analysis, which suggested the presence of reductive dehalogenases as functional biomarkers associated with OHR in the sediment samples. Specifically, PICRUSt analysis predicted the presence of potential genes of tetrachloroethene reductive dehalogenase and 3-chloro-4-hydroxyphenylacetate reductive dehalogenase, previously linked to OHR. Microcosm studies confirmed the dechlorination potential of tetrachloroethene to dichloroethene.

**Conclusion:**

This study demonstrates that River Nile sediment in industrialized area harbors distinct microbiomes enclosing various OHRB genera, providing substantial evidence for potential reductive dechlorination activity. It also provides potential functional biomarkers for OHR activity.

**Supplementary Information:**

The online version contains supplementary material available at 10.1186/s12866-025-03864-1.

## Introduction

Chloroethenes are group of persistent and toxic volatile organic compounds (VOCs) including tetrachloroethene (PCE), trichloroethene (TCE), cis-1,2-dichloroethene (cis-DCE), trans-1,2-dichloroethene (trans-DCE), 1,1-dichloroethene (1,1-DCE) and vinyl chloride (VC) [1, 2]. They pose a significant threat to environmental quality due to their widespread use in industrial processes and their tendency to contaminate soil and sediments [3, 4]. Due to their widespread usage and inappropriate disposal, chlorinated ethenes have been listed among the most ubiquitously distributed soil, groundwater and pollutants [5, 6]. Sediments, in particular, act as long-term reservoirs for these contaminants, potentially impacting aquatic ecosystems and human health through water column transport and bioaccumulation [7–10]. The long-term accumulation of chloroethenes can be carcinogenic to humans and impair their nervous and immune systems [11, 12].

Chlorinated ethenes tend to accumulate in anaerobic habitats including sedimentary environments causing pollution to ground water resources [13, 14]. Contamination of the River Nile by industrial wastes, agricultural runoff, and untreated sewage results in accumulation of their emerging pollutants in its sediments. Among the several remediation options for organohalide pollution, classical physical and chemical techniques can efficiently remove or degrade contaminants [15]. However, these approaches have challenges due to byproducts, high costs, and energy consumption. Bioremediation has emerged as a promising approach for the in-situ treatment of chloroethenes in sediments, offering a sustainable and cost-effective alternative to outdated methods.

Chloroethenes can be detoxified in a stepwise anaerobic biological process known as reductive dechlorination (RD) [16–19], in which highly chlorinated compounds are reduced into less chlorinated compounds and subsequently to ethene in the presence of H_2_ as electron donor [20]. Organohalide respiration (OHR) process has been well documented [21–25] and described as promising approach for detoxification of chloroethenes. OHR is carried out by membrane-associated reductive dehalogenase (RDase) enzymes [26–29], the majority of which have iron-sulfur (Fe-S) clusters and a corrinoid cofactor [22].

Organohalide respiring bacteria (OHRB) [30–34] use reductive dehalogenation to satisfy their energy needs for growth and metabolism. OHRB are widely distributed in a variety of habitats, including marine sediments, soils, and freshwater ecosystems, where they help to degrade naturally occurring organohalides produced by marine algae, fungi, and plants [35]. Their application in bioremediation process is a sustainable, cost-effective, and environmentally friendly method for in-situ remediation of organohalide pollution [36, 37].

Many microorganisms, such as *Dehalococcoides*, *Dehalobacter*, *Desulfitobacterium*, *Geobacter*, and *Sulfurospirillum* are capable of transforming organohalide contaminants and play an important part in bioremediation processes.

So far, OHRB have been described in three phyla, including *Proteobacteria*, *Firmicutes*, and *Chloroflexi* [38, 39]. Obligate OHRB with restricted metabolism; *Dehalococcoides mccartyi*, *Dehalogenimonas lykanthroporepellens* and *Dehalobacter* were described in *Chloroflexi* and *Firmicutes* [40, 41]. On the other hand, OHRB with versatile metabolic activities like *Geobacter*, *Anaeromyxobacter*, *Desulfomonile*, *Desulfuromonas*, *Desulfovibrio*, *Sulfurospirillum*, and *Desulfitobacterium* were described in *Proteobacteria* [42]. Among all known OHRB, strains from the genera *Dehalococcoides* and *Dehalogenimonas* have been shown to dechlorinate highly chlorinated ethenes, PCE and TCE, to ethene. Notably, reports indicate that *Dehalococcoides* can dechlorinate PCE to ethene [43], whereas *Dehalogenimonas* can dechlorinate TCE to ethene [44, 45].

Bioaugmentation with anaerobic dechlorinating bacteria has demonstrated effectiveness in treating deeper sediment layers contaminated with VC. However, the effectiveness of each approach depends on factors like site characteristics, contaminant type and concentration, microbial community composition (microbiome), and environmental conditions. The microbiome refers to the complex community of microorganisms inhabiting various environments, including our own bodies, soil, and sediments [46–48].

Advanced techniques like 16S rRNA gene amplicon sequencing, metagenomics, and proteomics can provide deeper insights into microbial communities and their degradation pathways, enabling targeted bioaugmentation strategies [49]. 16S rRNA gene amplicon sequencing of sediment is a powerful tool for unlocking the secrets hidden within the complex microbial communities that call these muddy depths home. By analyzing the collective genetic material of all the microbes present, we can gain insights into their diversity, function, and potential impact on the surrounding environment. Within these diverse communities, OHRB play a crucial role in maintaining environmental health by degrading persistent organohalides.

Sedimentary environments may represent a niche for a diverse group of microorganisms that could be exploited in bioremediation of a variety of environmental pollutants [50].

Studies show that heavily polluted marine sediments, despite contamination, host unique bacterial communities. Some of these bacteria have promising abilities to degrade various hydrocarbons [51, 52]. It has been reported that bacterial and archaeal communities in heavy metal-contaminated sediments displayed distinctive diversity and composition compared to their cleaner counterparts [53]. Previous studies confirmed the presence of a hydrocarbon-degrading microbiome in sedimentary environments, primarily adapted to anaerobic niches [54]. Characterization of the indigenous bacterial community within different sediments revealed a dominance of anaerobic strains known for their reductive dechlorination abilities [55–57].

Xu et al. [58] reported the dehalogenation of several organohalide contaminants by offshore marine sediment-enriched cultures dominated by *Dehalococcoides*. Therefore, it has been concluded that, highly contaminated aquatic sediments represent a potential reservoir of novel and diverse bacterial taxa with promising bioremediation capabilities [59, 60]. By gaining insights into the diversity and dynamics of the natural microbial populations in these benthic systems, we can develop more effective bioremediation strategies, leading to better environmental clean-up.

The present study was constructed to reveal the diversity of bacterial communities and to characterize the composition and functions of entire microbiomes in industrial site contaminated River Nile sediment. This helps identify native OHRB populations in River Nile sediment and assess their potential for bioremediation. OHR functional biomarkers prediction was used as substantial evidence for potential reductive dehalogenation processes. An integrated approach including 16S rRNA gene amplicon next generation sequencing and microcosm study was used to evaluate OHR potential in River Nile sediment. The overall outcome will be utilized in enrichment of specific OHRB strains from environmental samples to be used for targeted bioremediation applications.

## Material and methods

### Sediment sampling

Sediment samples, designated RNS1 and RNS2, were collected In October 2021, from two sites along the River Nile in Helwan, Egypt (RNS1 29° 46′ 13.8'' N 31° 17′ 25.0'' E; RNS2 29° 46′ 13.8'' N 31° 17′ 22.9'' E). These sites were chosen for their representation of the prevailing environmental pollution. Historical industrial activities and ongoing anthropogenic inputs from the surrounding densely populated area contribute to the contaminated conditions. Triplicate surface sediment samples were collected at each site using sterile polypropylene tubes. Samples were then immediately sealed and transported to the laboratory for subsequent analyses. Overall bacterial community structure and diversity in addition to identification of potential OHRB genera in sediment samples were examined by analysis of 16S rRNA gene amplicon sequences.

### Extraction genomic DNA and 16S rRNA gene amplification for illumina sequencing

Total genomic DNA was isolated from sediment samples collected from the PCE-enriched microcosms using the DNeasy PowerSoil Kit (QIAGEN, Hilden, Germany) following the manufacturer's instructions. Extracted DNA was subsequently stored at −20 °C for downstream molecular analyses. PCR amplification of the 16S rRNA gene targeting the bacterial community was performed using the universal primer pair 341F (CCTACGGGNGGCWGCAG) and 805R (GACTACHVGGGTATCTAATCC). This primer set specifically amplifies the hypervariable region V3-V4 of the bacterial 16S rRNA gene. The PCR reaction was outsourced to Macrogen (Seoul, Korea) and conducted according to Illumina's 16S rRNA Gene Amplicon Sequencing Library protocols (Illumina website: https://www.illumina.com). The thermal cycling program consisted of an initial denaturation step at 95 °C for 3 min, followed by 25 amplification cycles of 95 °C for 30 s, 55 °C for 30 s, and 72 °C for 30 s. A final extension step was included at 72 °C for 5 min.

### Illumina Miseq sequencing and bioinformatics analysis

Following PCR amplification, amplicons targeting the V3-V4 region of the 16S rRNA gene (~ 450 bp) were purified and sequenced by Macrogen (Daejeon, Korea) on an Illumina MiSeq platform using a 2 × 300 paired-end read strategy. Subsequently, the EZ-biocloud server (https://help.ezbiocloud.net/ezbiocloud-16s-database/) and its internal PKSSU4.0 reference database were employed for operational taxonomic unit (OTU) picking and taxonomic assignment of sequences from the phylum to the genus level, with a particular focus on identifying OHRB sequences. Detailed bioinformatics analysis involved joining forward and reverse reads using VSEARCH v2.13.4 [61] after uploading them to the EZ-biocloud server. Chimeric sequences, low-quality reads (Q-score < 25), and potential PCR artifacts were subsequently filtered out. OTU clustering was then performed at a 97% sequence similarity threshold using both UCLUST [62] and CD-HIT [63] software.

### Community analysis of microbiomes: diversity, predicted function, and phylogeny

Alpha diversity metrics, including Chao1 richness estimator for good coverage, rarefaction curves, Shannon diversity index, and Simpson's diversity index, were calculated using the EZ-biocloud server. Venn diagram( at the genus level) was generated using the InteractiVenn online tool (https://www.interactivenn.net/) to visualize the shared and unique OTUs across samples, considering their relative abundance values. Functional prediction of key biomarkers based on taxonomic 16S rRNA gene data was performed using the Phylogenetic Investigation of Communities by Reconstruction of Unobserved States (PICRUSt) tool integrated within the EZ-biocloud server [64]. Heatmaps depicting predicted functional metabolic activities, including OHR and others, were constructed using the online tool SRplot (https://www.bioinformatics.com.cn/en?keywords=heatmap). Finally, phylogenetic relationships among the identified genera were inferred using the neighbor-joining method implemented in MEGA11 software. The resulting phylogenetic tree was visualized using the Interactive Tree of Life (ITOL) tool (https://itol.embl.de/).

### Targeted PCR detection of Dehalococcoides and Dehalococcoides genera in sediment

A PCR-based approach was employed for the targeted detection of genera *Dehalococcoides* and *Dehalogenimonas* in the sediment samples. Specific primer sets 1F (GATGAACGCTAGCGGCG) and 259R (CAGACCAGCTACCGATCGAA), 631F (CGTCATCTGATACTGTTGGACTTGAGTATG) and 769R (ACCCAGTGTTTAGGGCGTGGACTACCAGG) were designed to amplify the 16S rRNA genes of *Dehalococcoides* and *Dehalogenimonas*, respectively. PCR primer specificity validation data are present (Table S1). The PCR reaction conditions were identical to those previously described in the thermal cycling protocol [65–67].

### Establishment of PCE-dechlorinating enrichment culture

River Nile sediment collected from Cairo, Egypt, was used as the inoculum for enriching OHRB in this study. Approximately 10 g of sediment were aseptically added to 100 mL anaerobic bottles containing 40 mL of basal mineral salts medium. The medium composition (g/L) was: 2.5 NaHCO3, 1 NaCl, 0.5 MgCl₂·6H₂O, 0.2 KH₂PO₄, 0.3 NH₄Cl, 0.3 KCl, 0.15 CaCl₂·2H₂O, 0.48 Na₂S₂O₃·5H₂O, 5 mM sodium acetate, 1 mL trace element solution (1000 ×), and 1 mL vitamin solution (1000 ×) [68]. Bottles were sealed with Teflon-coated butyl rubber septa and aluminum crimp caps, purged with hydrogen gas for 30 min to establish anoxic conditions, and subsequently spiked with PCE as the electron acceptor. Incubations were carried out at 28 °C for one month. Headspace gas composition was monitored weekly using gas chromatography coupled with a flame ionization detector (GC-FID) as described below.

### GC-FID analysis of PCE and daughter products in enrichment cultures

PCE and its less chlorinated daughter products, including TCE, DCE, VC, and ethene, were analyzed by using GC-FID. Briefly, 100 μL of headspace gas from the PCE-enriched cultures were withdrawn using a gas-tight syringe and injected directly into an Agilent 7890 GC-FID system (Agilent Technologies, Santa Clara, CA, USA). The analytical separation employed hydrogen as the carrier gas at a constant flow rate of 2 mL/min. The following temperature program was used: initial hold at 50 °C for 5 min, followed by a ramp at 20 °C/min to a final temperature of 240 °C, which was held for 0 min. The injector and detector temperatures were maintained at 240 °C and 300 °C, respectively.

## Results

### Bacterial community compositions of River Nile sediments

**Fig. 1:**
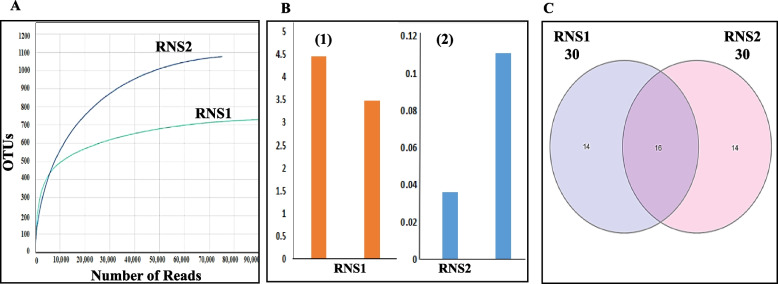
Alpha (**A** and **B**) and Beta (**C**) diversity indices of RNS1 and RNS2 microbiomes investigated in this work. **A** Rarefaction curves, B1-Shannon diversity index, B2-Simpson diversity index, and (**C**) Venn diagram displaying the shared and unique OTUs for the two microbiomes addressed in this study

Illumina MiSeq sequencing of 16S rRNA gene amplicon was employed to characterize the taxonomic composition of bacterial communities in two sediment samples. This approach revealed the microbiome structure of each sample. The sequencing yielded valid read percentages of 89.7% and 75.3% for RNS1 and RNS2 microbiomes, respectively (Table 1). These reads corresponded to 729 and 1077 operational taxonomic units (OTUs) in RNS1 and RNS2, respectively (Table 1). Good's coverage values exceeded 99% for both microbiomes, indicating that the sequencing captured and identified the majority of bacterial populations present (Table 1). Rarefaction curves (Fig. 1A) reached a plateau, confirming adequate sequencing depth for comprehensive analysis of the microbial communities. The observed Shannon and Simpson diversity indices (Fig. 1B (B1&B2)) suggested a higher level of bacterial diversity in the RNS1 sample compared to RNS2. Venn diagram analysis (Fig. 1C) revealed 16 OTUs shared by both microbiomes, while 14 OTUs were unique to each sample.

**Table 1:** Numbers of sequences, OTUs and Good's coverage of two microbiome obtained in this sudy

Sample name	Sequence reads	OTUs	Goods coverage of library(%)
RNS1	89,763	729	100.0
RNS2	75,251	1077	99.9

**Fig. 2:**
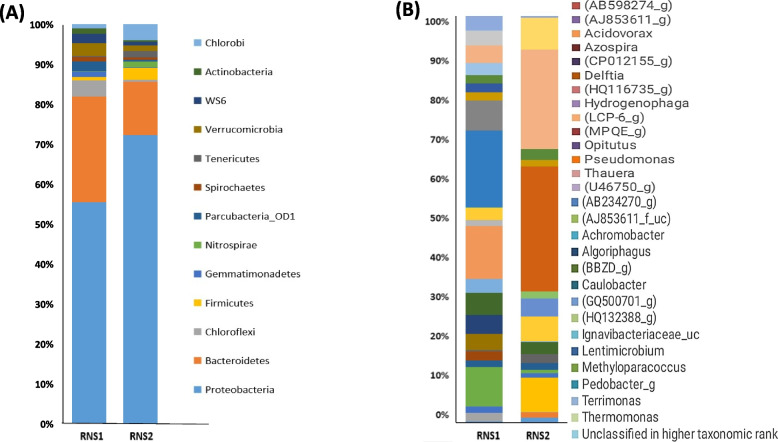
Bacterial community composition at (**A**) the phylum level and (**B**) genus level

Taxonomic affiliation of raw 16S rRNA gene sequences at the phylum level (Fig. 2A) revealed *Proteobacteria* as the dominant phylum in both RNS1 (51.4%) and RNS2 (71.1%) sediment samples. *Bacteroidetes* (24.5%), *Chloroflexi* (3.79%), *Verrucomicrobia* (2.92%), *Parcubacteria* (2.26%), WS6 (2.18%), and *Gemmatimonadetes* (1.26%) were the next most abundant phyla in RNS1, while RNS2 exhibited a distinct composition with *Bacteroidetes* (13.1%), *Chlorobi* (3.9%), *Firmicutes* (2.88%), *Tenericutes* (1.57%), *Nitrospirae* (1.45%), and *Verrucomicrobia* (1.39%) following *Proteobacteria*. At the genus level (Fig. 2B), RNS1 displayed *Lentimicrobium* (12.9%) as the most abundant genus, followed by *Methylophilaceae* (HQ116735_g) (8.9%), *Acidovorax* (6.6%), *Methyloparacoccus* (4.97%), *Delftia* (3.67%), and *Caulobacter* (3.17%). In contrast, RNS2 was dominated by *Chromatiales* (MPQE_g) (25.5%), with *Thauera* (20.3%), *Bacteroidales* (AJ853611_g) (7.1%), *Thiobacillaceae* (U46750_g) (6.5%), and *Hydrogenophaga* (5.1%) as the subsequent most abundant genera.

### Identification of putative obligate and non-obligate OHRB genera in sediment

**Fig. 3:**
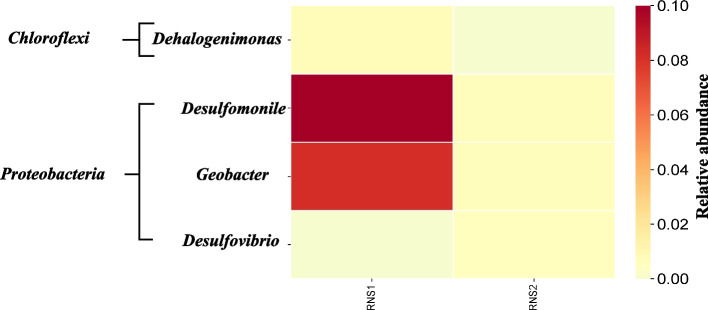
Heatmap showing the identification and distribution of various potential obligate and non-obligate OHRB genera throughout the RNS1 and RNS2 microbiomes addressed in this study

**Fig. 4:**
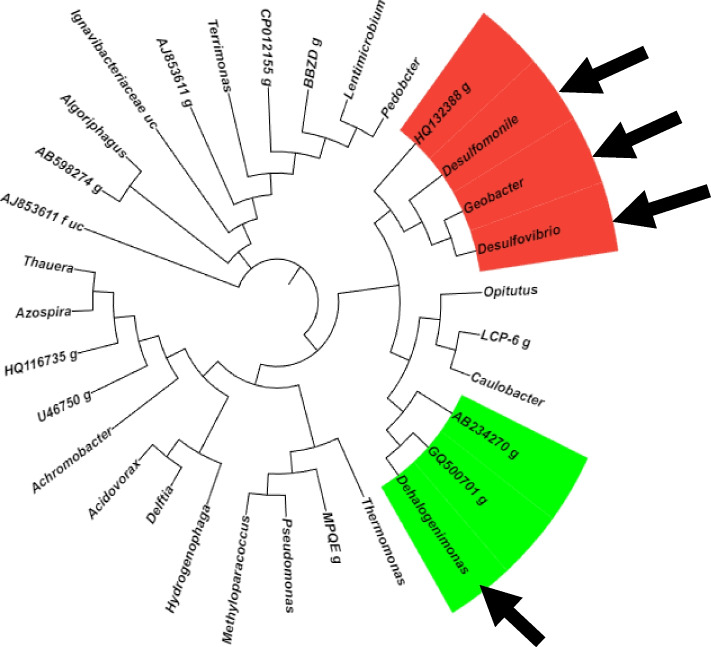
16S rRNA-based circular phylogenetic tree showing all genera identified in this study. Genera belonging to phyla Chloroflexi and Proteobacteria, identified to harbor OHRB, were highlighted in the green and red color, respectively. Black arrows represent the genera of OHRB identified in the study

**Fig. 5:**
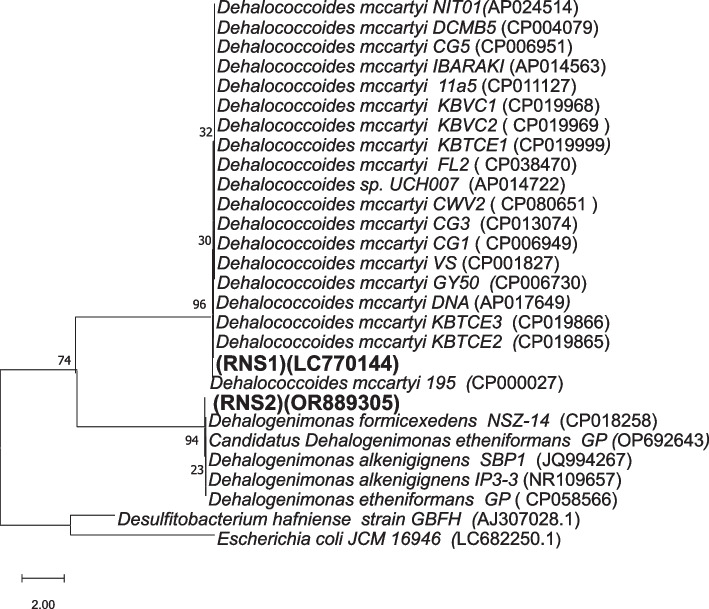
A neighbor-joining tree based on 16S rRNA gene sequences demonstrating the phylogenetic relationship of RNS1 and RNS2, obtained in this investigation and displayed in bold font, with members of the genus Dehalogenimonas, Dehalococcoides mccartyi strains

Analysis of 16S rRNA gene amplicon sequences revealed the presence and distribution of several potential obligate and non-obligate OHRB genera within the RNS1 and RNS2 microbiomes (Fig. 3). These included *Dehalogenimonas* (*Chloroflexi*), *Geobacter*, *Desulfomonile*, and *Desulfovibrio* (*Proteobacteria*). Notably, *Desulfomonile* and *Geobacter* were found in both samples, while *Dehalogenimonas* was specific to RNS1 and *Desulfovibrio* was exclusive to RNS2. Figure 4 provides a visual representation of these findings. To further investigate the presence of specific OHRB genera, PCR amplification was conducted using primer sets designed to target the 16S rRNA genes of *Dehalococcoides* (Fig. S1) and *Dehalogenimonas* (Fig. S2). Gel electrophoresis confirmed the presence of the expected amplicons at the appropriate sizes: 258 bp for *Dehalococcoides* and 194 bp for *Dehalogenimonas*. Subsequent direct sequencing of these PCR products, followed by BLAST analysis against the NCBI database and phylogenetic tree construction (Fig. 5), validated the presence of both *Dehalococcoides* and *Dehalogenimonas* in the sediment samples.

### Functional biomarkers for OHR potential and diverse anaerobic metabolism

**Fig. 6:**
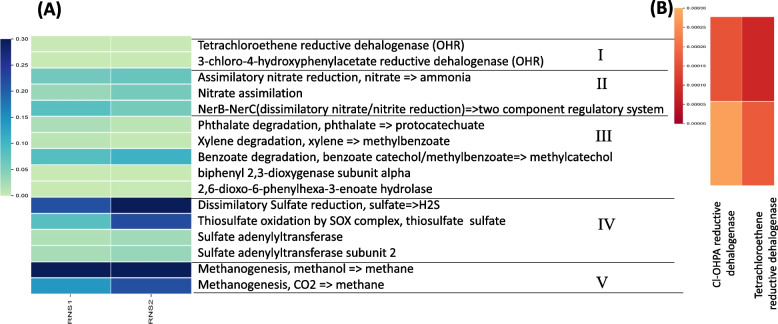
PICRUSt analysis based on 16S rRNA gene sequences obtained in this study, where (**A**) represents the major metabolic biomarkers identified as I, OHR; II, Nitrogen cycle; III, Aerobic hydrocarbon degradation; IV, sulfur cycle; and V, Methanogenesis, and (**B**) heatmap showing the two reductive dehalogenases biomarkers for OHR

PICRUSt functional prediction analysis, based on 16S rRNA gene sequencing data, identified PCE reductive dehalogenase (KEGG KO K21647) and 3-chloro-4-hydroxyphenylacetate (Cl-OHPA) reductive dehalogenase (KEGG KO K21566) as potential biomarkers for OHRB genera within the sediment samples (Fig. 6A, B). These findings, coupled with the identification of known OHRB genera through taxonomic analysis (Fig. 3) and the successful PCR amplification of *Dehalococcoides* (Fig. S1) and *Dehalogenimonas* 16S rRNA genes (Fig. S2), suggest the potential for OHR activity in the studied sediments. Additionally, PICRUSt revealed the presence of functional biomarkers indicative of diverse metabolic pathways across the samples (Fig. 6A). Notably, predicted biomarkers for anaerobic processes, such as methanogenesis and dissimilatory sulfate reduction, exhibited high relative abundances. These observations suggest a functionally diverse microbial community within the River Nile sediments, with a particular emphasis on anaerobic capabilities.

### Confirmation of dechlorination activity in sediment

The potential for OHR and bioremediation in the sediment samples was further evaluated using enrichment cultures spiked with PCE as a model organohalide contaminant. PCE was chosen due to its frequent detection as a groundwater pollutant and its established toxicity. After one month of incubation, GC-FID analysis revealed the dechlorination of PCE to its daughter products, TCE and DCE (Fig. S3&S4). These findings provide additional evidence for the presence and activity of dechlorinating microorganisms within the sediment samples.

## Discussion

Organohalide pollutants pose a significant threat to human health and the environment [69]. Anaerobic reductive dehalogenation by OHRB is a well-established bioremediation mechanism that reduces the toxicity and enhances the biodegradability of these contaminants [70]. River sediments in urban areas polluted with organohalides may provide ideal niches for OHRB growth [71]. This study aimed to assess the potential OHR activity in sediment samples using PCE as a model contaminant, and to investigate the presence of known OHRB genera and their predicted reductive dehalogenase genes via different approaches.

Enrichment culture experiments revealed stable PCE-to-DCE dechlorination activity in the sediment samples. Analysis of Illumina amplicon sequencing data of 16S rRNA genes from the active enrichment cultures identified four OHRB genera: *Dehalogenimonas, Geobacter, Desulfovibrio*, and *Desulfomonile* [31]. Thereafter, PCR with specific primer sets targeting 16S rRNA genes identified the existence of *Dehalococcoides* and confirmed the existence of *Dehalogenimonas* [65–67]. The identified OHRB genera can be classified into obligate (*Dehalococcoides* and *Dehalogenimonas*) and non-obligate (*Geobacter*, *Desulfovibrio*, and *Desulfomonile*) OHRB [32]. Identification of obligate OHRB imply actual OHR activity, as the OHR is the sole route for energy conservation and growth known so far in these microorganisms.

Also, it was suggested that the non-obligate genera *Desulfovibrio*, *Desulfomonile*, and *Geobacter* potentially involved because previous studies suggested that certain strains of these genera were identified with OHR activity. Previous research has shown that *Desulfomonile* dechlorinates PCE and 3-chlorobenzoate [31, 72], *Geobacter lovleyi* strain SZ dechlorinates PCE and TCE [73], and *Desulfovibrio* dechlorinates 2-chlorophenol and 2,6-dichlorophenol [31].

While *Dehalococcoides, Dehalobacter, Desulfitobacterium, Geobacter, Sulfurospirillum*, and *Desulfuromonas* have been documented to dechlorinate PCE [31], the role of *Desulfovibrio, Desulfomonile*, and *Dehalogenimonas* identified in this study requires further investigation. Limited literature exists demonstrating PCE dechlorination activity for *Desulfovibrio* and *Desulfomonile*, with documented activity focused on other organohalides [31]. Similarly, *Dehalogenimonas* has only been shown to dechlorinate TCE, not PCE [44]. These findings suggest a lower likelihood that these three genera were directly involved in PCE dechlorination within our enrichment cultures. In contrast, *Dehalococcoides*, a well-established PCE dechlorinator [74], was identified in our samples. Notably, some studies report *Dehalococcoides* demonstrating PCE-to-DCE dechlorination activity alongside its ability to dechlorinate the recalcitrant pollutant PCB [75, 76]. This observation raises the possibility of PCB dechlorination activity in our sediment samples as well. However, to definitively identify the genera responsible for PCE dechlorination in our enrichment cultures, more quantitative approaches using quantitative real-time PCR (RT-qPCR) or fluorescence in situ hybridization (FISH) analysis are warranted. It has been previously shown that OHRB can couple dehalogenation with growth or increased biomass in mixed cultures. This association was confirmed through RT-qPCR analysis with specific OHRB primers [77–79] and FISH analysis, which targeted OHRB cells using specific probes [79–81].

Previous studies have demonstrated dechlorination activity in Nile River sediments [82] reporting the transformation of chlorinated phenols to less chlorinated derivatives. Using PCR-DGGE analysis of 16S rRNA genes, this study identified *Geobacter*, *Pseudomonas*, *Desulfitobacterium*, *Desulfovibrio*, and *Clostridium* as potential bacterial genera involved in chlorophenol dechlorination [82]. Additionally, [78] described a dechlorinating microcosm established from Arako River sediment in Japan, exhibiting PCE dechlorination to ethene. Their study identified *Dehalococcoides* as the responsible dechlorinator through qPCR and whole metagenome analyses.

PICRUSt analysis approach, based on 16S rRNA gene sequencing data, predicted the presence of functional biomarkers for potential OHR activity, specifically reductive dehalogenases. PICRUSt is a well-established tool for identifying potential functional capabilities within microbial communities based on taxonomic information [64]. Prediction of dehalogenases genes using PICRUSt has been reported recently [83]. The PICRUSt prediction was also made in a study conducted by Ghandehari et al. [84], however the identified enzymes may not necessarily represent the classical reductive dehalogenases used by OHRB.

Beyond OHR-related biomarkers, PICRUSt revealed the presence of functional markers for diverse metabolic pathways, with some, such as methanogenesis and dissimilatory sulfate reduction, exhibiting high relative abundances. These activities are typically associated with strict anaerobic conditions, suggesting a strong potential for anaerobic metabolism, particularly OHR activity, within the sediment microbial community. Notably, a previous study investigating chlorobenzene bioremediation under anaerobic conditions using Nile River sediment as inoculum also reported the presence of methanogenesis and dissimilatory sulfate reduction [85]. However, it is important to distinguish that the previous study utilized chlorobenzenes as electron donors, while our study focuses on chloroethenes as electron acceptors.

Overall, environmental parameters such as soil moisture, temperature, pH, organic matter concentration, nutrient availability, and nutritional variables such as soil C:N ratios influence the structure and activity of microbial communities [86]. Previous studies suggest the dechlorination of PCE or TCE contaminants have been carried out at temperatures ranging from 20 °C and 38 °C [87, 88]. Furthermore, *Dehalogenimonas* strain GP cells incubated at 30 °C and 25 °C exhibited the highest rates of VC dechlorination [44].

## Conclusion

This study identified two microbiomes from River Nile sediments with potential for PCE reductive dechlorination. Analysis of 16S rRNA gene amplicon sequences detected several potential OHRB, suggesting their potential role in bioremediation. Functional prediction using PICRUSt showed the presence of PCE reductive dehalogenase and 3-Cl-OHPA reductive dehalogenase, potential biomarkers for OHR. Our findings imply that the suggested OHRB genera in the sediment samples are potentially responsible for PCE dechlorination within our established enrichment cultures.

## Supplementary Information

Supplementary Material 1.

Supplementary Material 2.

Supplementary Material 3.

## Data Availability

Obtained sequences of 16S rRNA genes for genera Dhc and Dhg were submitted into NCBI GenBank database under accession numbers LC770144 and OR889305, respectively. Raw amplicon 16S rRNA data were submitted to NCBI SRA under accession number PRJNA1089236.

## Abbreviations

VOCs: Volatile Organic Compounds
PCE: Tetrachloroethene
TCE: Trichloroethene
DCE: Dichloroethene
VC: Vinyl chloride
PCB: Polychlorinated biphenyls
RD: Reductive dechlorination
RDase: Reductive dehalogenase enzyme
OHR: Organohalide respiration
OHRB: Organohalide respiring bacteria
OTUs: Operational taxonomic units
PICRUSt: Phylogenetic Investigation of Communities by Reconstruction of Unobserved States
ITOL: Interactive Tree of Life
GC-FID: Gas chromatography with flame ionization detection
PceA: Tetrachloroethene reductive dehalogenase
Cl-OHPA: 3-Chloro-4-hydroxyphenylacetate reductive dehalogenase
RT-qPCR: Quantitative real time PCR
FISH: Fluorescence in situ hybridization
