# Supplementary material for: Unveiling organohalide respiration potential in River Nile sediments via 16S rRNA gene amplicon sequencing of endogenous bacterial communities
Source: BMC Microbiol. 2025 Mar 31;25:186. doi: 10.1186/s12866-025-03864-1 (PMC11956321; doi:10.1186/s12866-025-03864-1)
Supplement: Supplementary file 1 — Supplementary Material 1. [file 12866_2025_3864_MOESM1_ESM.pptx]

## Slide 1
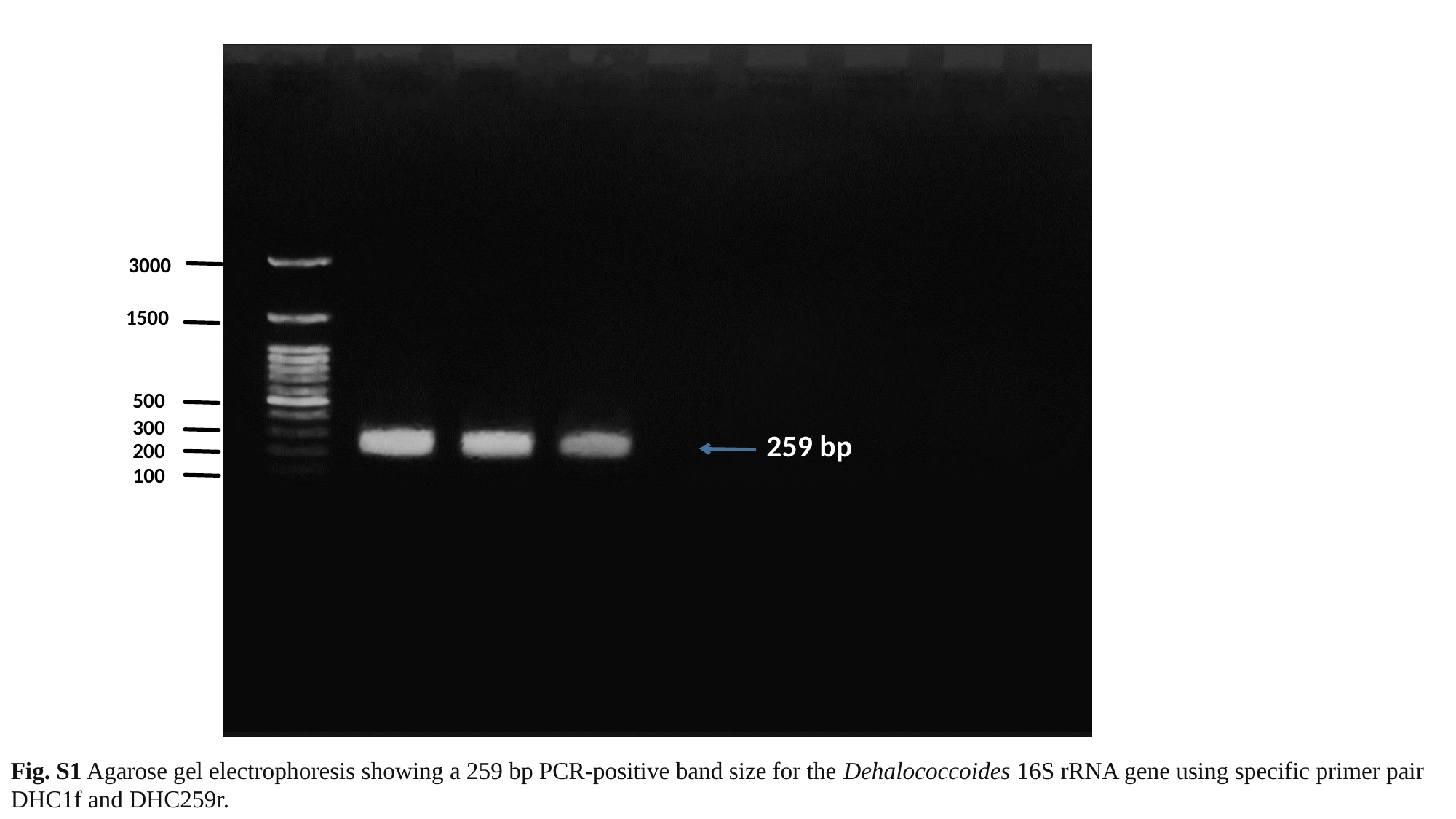

3000
 1500
 500
 300
259 bp
 200
 100
Fig. S1 Agarose gel electrophoresis showing a 259 bp PCR-positive band size for the Dehalococcoides 16S rRNA gene using specific primer pair DHC1f and DHC259r.

## Slide 2
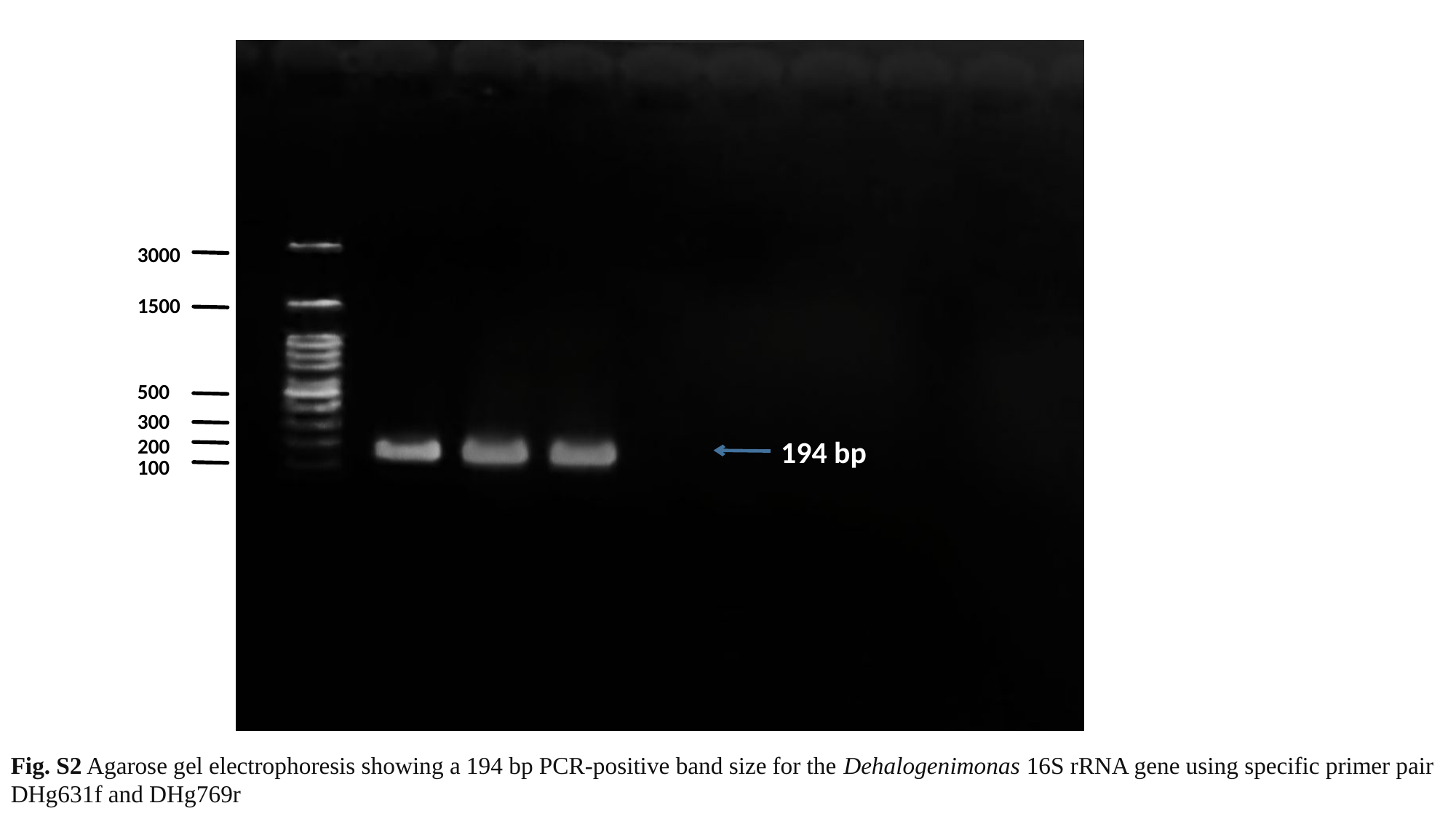

3000
 1500
 500
 300
 200
194 bp
 100
Fig. S2 Agarose gel electrophoresis showing a 194 bp PCR-positive band size for the Dehalogenimonas 16S rRNA gene using specific primer pair DHg631f and DHg769r

## Slide 3
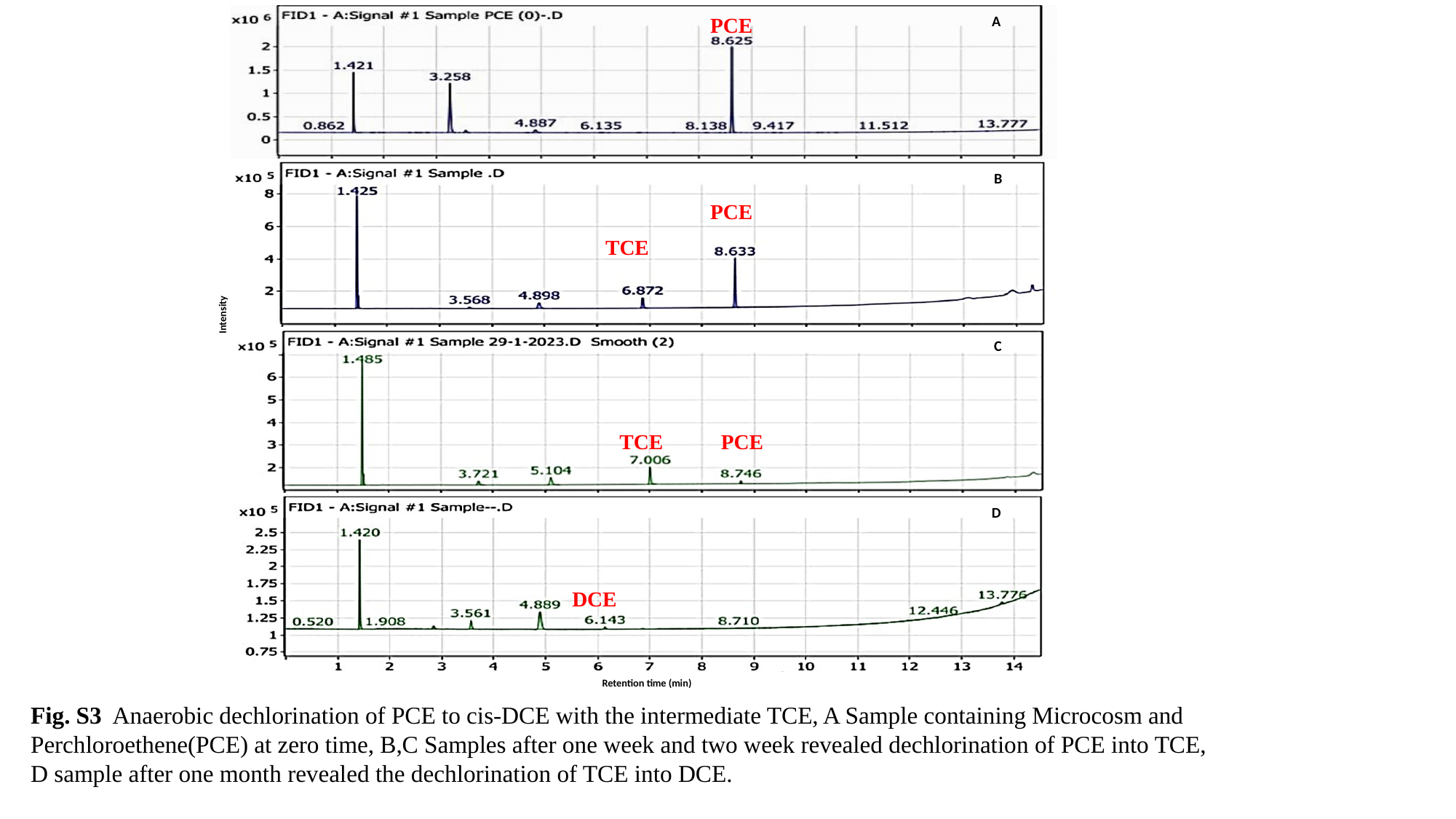

A
B
Intensity
C
D
Retention time (min)
PCE
PCE
TCE
PCE
TCE
DCE
Fig. S3 Anaerobic dechlorination of PCE to cis-DCE with the intermediate TCE, A Sample containing Microcosm and Perchloroethene(PCE) at zero time, B,C Samples after one week and two week revealed dechlorination of PCE into TCE, D sample after one month revealed the dechlorination of TCE into DCE.

## Slide 4
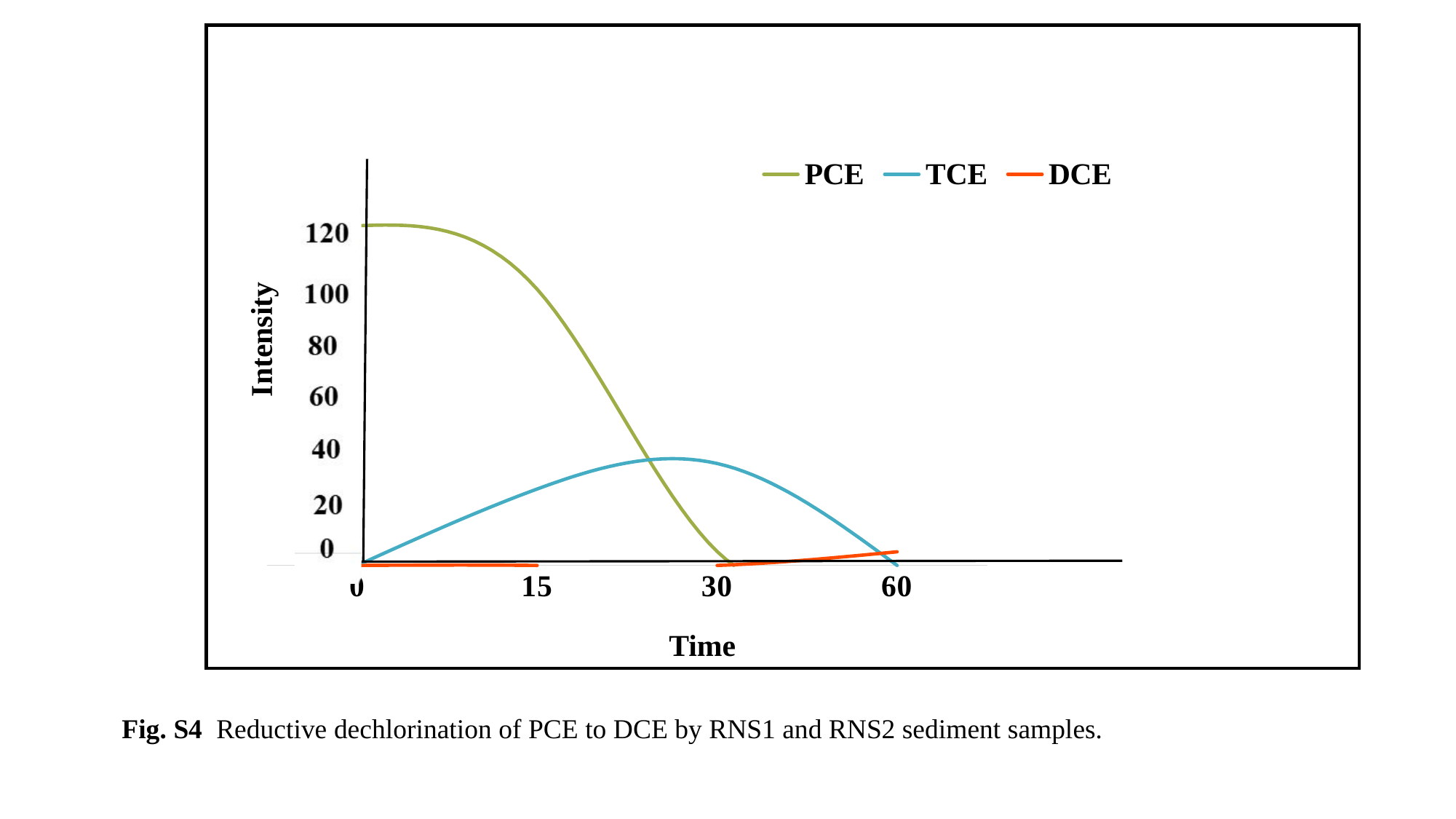

### Chart
| Category | PCE | TCE | DCE |
|---|---|---|---|
| 0 | 100.0 | 0.0 | 0.0 |
| 15 | 81.3 | 22.5 | 0.0 |
| 30 | 4.1 | 30.0 | 0.0 |
| 60 | 0.0 | 0.0 | 4.0 |
Intensity
Time
Fig. S4 Reductive dechlorination of PCE to DCE by RNS1 and RNS2 sediment samples.

## Slide 5
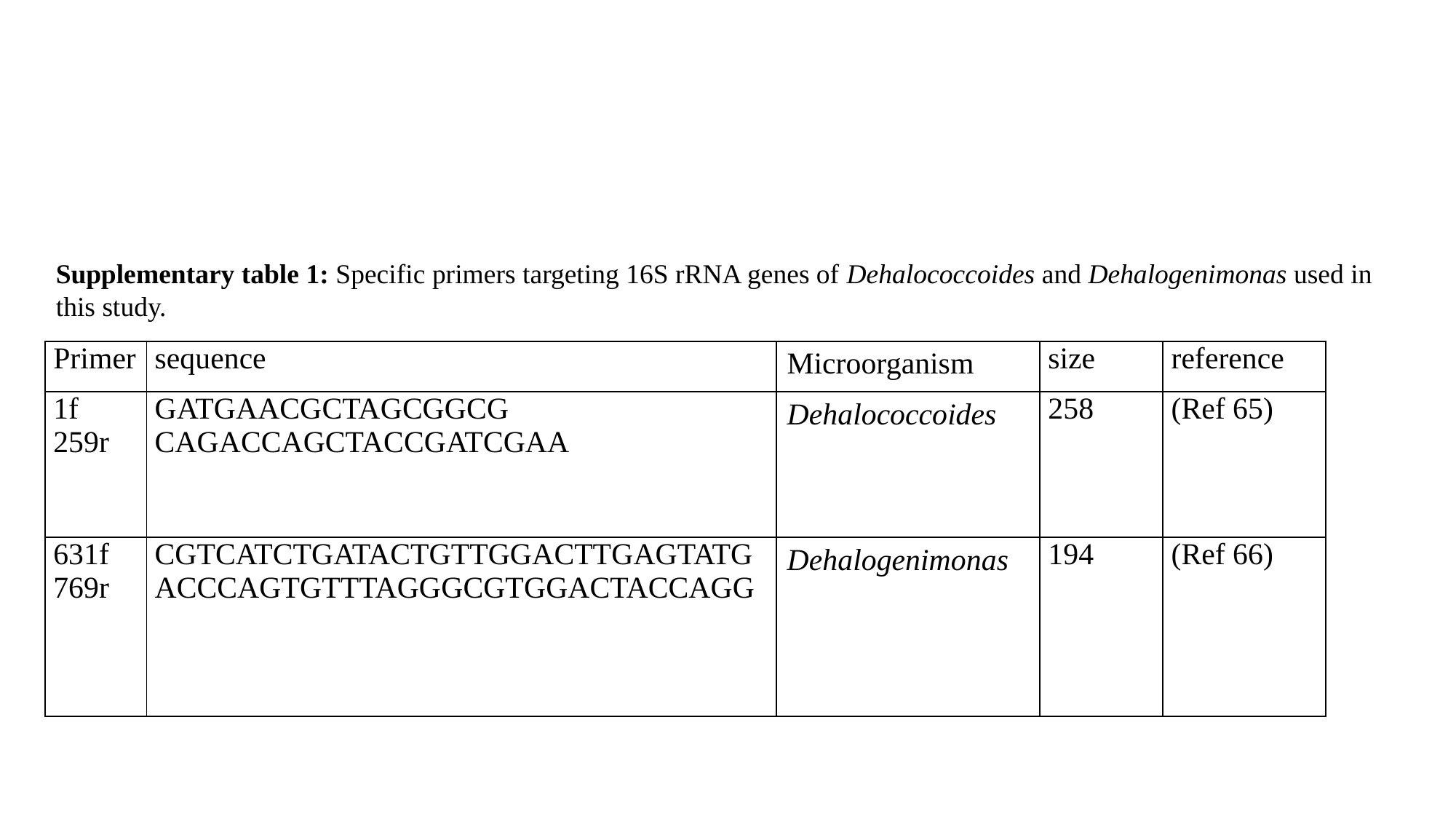

Supplementary table 1: Specific primers targeting 16S rRNA genes of Dehalococcoides and Dehalogenimonas used in this study.
| Primer | sequence | Microorganism | size | reference |
| --- | --- | --- | --- | --- |
| 1f 259r | GATGAACGCTAGCGGCG CAGACCAGCTACCGATCGAA | Dehalococcoides | 258 | (Ref 65) |
| 631f 769r | CGTCATCTGATACTGTTGGACTTGAGTATG ACCCAGTGTTTAGGGCGTGGACTACCAGG | Dehalogenimonas | 194 | (Ref 66) |
